# Association between carbohydrate intake and lung function in American adults: The National Health and Nutrition Examination Survey (NHANES) 2007 to 2012

**DOI:** 10.1097/MD.0000000000045669

**Published:** 2025-10-31

**Authors:** Yin Xu, Xinmei Wang, Guofeng Wang, Wei Wei, Ning Li

**Affiliations:** a Department of Gerontology, The 960th Hospital of PLA, Jinan, China; b Department of Pharmacy, The 960th Hospital of PLA, Jinan, China.

**Keywords:** carbohydrate intake, COPD, FEV1, FVC, lung function

## Abstract

The association between lung function and dietary intake of carbohydrates remains unclear. In this study, we aimed to evaluate the association between the type and amount of carbohydrate intake and glucose metabolism on the forced expiratory volume 1 and forced vital capacity. The lung function parameters, carbohydrate, dietary intake, and the 2-hour glucose were extracted from the National Health and Nutrition Examination Survey. Multivariate regression analysis was used to investigate the correlation between the variables according to gender and age. Carbohydrates and dietary fiber intake were positively associated with forced vital capacity and forced expiratory volume 1. Conversely, the 2-hour glucose was negatively associated with lung function. The association between carbohydrates and lung function was more significant in men and individuals above the age of 40 years. Optimal carbohydrate intake and good glucose metabolism can improve lung function. These findings could be used to develop dietary recommendations to reduce the risk of developing lung disease.

## 1. Introduction

Respiratory disease is the 2nd leading cause of death globally. Chronic obstructive pulmonary disease (COPD) is the most common respiratory disease and is characterized by limited airflow within the lung.^[1]^ Patients with the most severe airflow obstruction risk dying of respiratory failure.^[2]^ Epidemiological surveys have reported that the diagnosed prevalence of respiratory diseases may be much lower than the actual prevalence. Therefore there is a need to identify patients at risk of developing COPD.^[3,4]^

Pulmonary function is a clinical assessment that quantitatively evaluates lung volumes, capacities, rates of flow, and gas exchange to determine respiratory function and diagnose potential pulmonary disorders such as COPD, asthma, and pulmonary fibrosis. It can also be used to predict the risk of mortality in patients.^[5,6]^ The forced vital capacity (FVC), the forced expiratory volume in 1 second (FEV1), and the forced expiratory flow rate (FEF) are common metrics used to assess lung function.^[7–10]^ FVC is the total amount of air that can be forcefully exhaled from the lungs after taking the deepest breath possible. FEV1 is the volume of air that can be forcefully exhaled in the first second of a breath while FEF measures the airflow rate during the middle portion of a forced expiration, typically measured between 25% and 75% of the FVC.

The relationship between dietary nutrition and disease, especially chronic disease, has been the focus of disease prevention research.^[11,12]^ Carbohydrates are organic molecules composed of carbon, hydrogen, and oxygen that play a crucial role in cellular function and metabolic processes. Carbohydrates can be divided into simple carbohydrates and complex carbohydrates according to their structure. Simple carbohydrates include monosaccharides such as glucose, fructose, and galactose and disaccharides such as maltose, sucrose, and lactose. Complex carbohydrates include starch, glycogen, and dietary fiber. A diet low in vitamins and fruits, and high in fat was linked with an increased risk of developing COPD.^[13]^ Recent studies have shown a correlation between the intake of carbohydrates and the development of COPD. A diet rich in dietary fiber was linked with a lower risk of developing COPD.^[14]^ Conversely, a diet rich in fructose was found to increase the risk of developing lung disease.^[15]^ More research is therefore required to assess the impact of carbohydrate intake and lung disease.

This study aimed to evaluate the effects of carbohydrate intake on lung function parameters such as FVC, FEV1, and FEF by analyzing the data of the National Health and Nutrition Examination Survey (NHANES) conducted in the United States between 2007 and 2012. The findings of this study could be used to develop strategies to prevent and treat lung disease.

## 2. Methods

### 2.1. Study population

This study analyzed data from 2 cycles of the NHANES performed between 2007 and 2012. The method used to collect the data in this survey is described on the NHANES website (https://wwwn.cdc.gov/nchs/nhanes/Default.aspx). The participants age below 20 years, without baseline data on carbohydrates, total sugars, and dietary fiber intake as well as spirometry data were excluded.

### 2.2. Dietary data

The intake of food and beverages consumed within 24 hours before the interview (midnight–midnight) was used to estimate the intake of carbohydrates. According to the type of food consumed the carbohydrates were divided into carbohydrates, total sugar, and dietary fiber.

### 2.3. Spirometry

The FVC, FEV1, FVC%, FEV1%, and FEV1/FVC measurements were acquired from the spirometry tests.

### 2.4. Covariates

All covariates were selected based on single-factor analysis and previous studies.^[16–19]^ Participants were classified as having a chronic disease if they reported ever suffering from angina, congestive heart failure, coronary heart disease, type 2 diabetes mellitus, and/or stroke in the personal interview. The participants were divided into 3 groups based on their smoking habits: never smoked, current smokers, and former smokers. The participants were classified as never smoked if they had consumed fewer than 100 cigarettes in their lifetime. The current smokers group included individuals who smoked at least 100 cigarettes in their lifetime and were still smoking, while the former smokers group included individuals who smoked at least 100 cigarettes but had since quit smoking.^[20]^ Participants who consumed 12 alcoholic beverages per year were identified as alcohol drinkers. During the health survey, all participants had their blood pressure measured 3 times consecutively by a medical professional using a sphygmomanometer. The 3 repeated measurements were averaged. Participants with a least 3 systolic blood pressure of 140 mm Hg or higher and/or diastolic blood pressure of 90 mm Hg or higher or who have been informed by a healthcare professional that they had high blood pressure were identified as suffering from hypertension. Participants who have been told by a health professional that they had type 2 diabetes mellitus or hemoglobin A1c levels of 6.5% or higher were classified as type 2 diabetic. The physical activity questionnaire was used to obtain the physical activity data of participants, in which work activities and recreational activities were classified as intense, moderate and other according to their intensity.

### 2.5. Statistical analyses

The data were weighted according to NHANES requirements to be representative of the general population in the United States. All analyses were performed using the EmpowerStats statistical software (X&Y Solutions, Boston) and R software version 4.3.2. Continuous variables were expressed as means ± standard deviations and minimum/maximum values, and categorical variables were expressed as percentages. The carbohydrate intake values were divided into 2 quartiles based on median (high carbohydrate intake:>1.30 g; low carbohydrate intake:≤1.30 g). Multiple regression analysis adjusted for covariates was used to analyze the relationship between the relationship between the 3 types of carbohydrates with FVC, FEV1, FVC%, FEV1%, and FEV1/FVC. The relationship between the intake of carbohydrates, total sugar, dietary fiber, and spirometry was analyzed using spline smoothing and a generalized additive mixed model (GAMM). In order to further evaluate the relationship between glucose tolerance and lung function, fasting blood glucose and 2-hour post-meal blood glucose data were included in the analysis. Subgroup analyses according to sex and age were also performed. For all statistical tests, a *P*-value below .05 was considered statistically significant.

## 3. Results

### 3.1. Participant characteristics

A total of 477 participants were included in the study. The mean age of the participants was 47.91 ± 15.59 years. The clinical characteristics of the study population according to their carbohydrate intake are shown in Table 1. The high carbohydrate group had a significantly higher number of males, non-Hispanic white, more educated, and nonsmokers than those with a low carbohydrate intake. Moreover, the high carbohydrate quartile group tended to consume more energy, sugar and dietary fiber, and had significantly higher FVC, FEV1, FEV1%, and FEV1/FVC levels (*P* < .05). Conversely, the participants in the high carbohydrate group had lower fasting glucose and 2-hour glucose levels (*P* < .05).

**Table 1:** Characteristics of the participants.

	Low carbohydrate intake	High carbohydrate intake	*P*-value
Age (yr, mean ± SD)	46.39 ± 14.81	46.22 ± 14.28	.8955
PIR (%)	3.54 ± 1.52	3.77 ± 1.46	.1063
Body mass index (kg/m^2^)	26.90 ± 5.97	27.17 ± 6.53	.629
Gender (%)			.0003
Male	26.7	42.46	
Female	73.3	57.54	
Race/ethnicity (%)			.6662
Mexican American	3.15	1.84	
Other Hispanic	3.64	2.91	
Non-Hispanic White	78.94	83.3	
Non-Hispanic Black	6.67	4.4	
Other race	7.6	7.55	
Education level (%)			.2717
Less than high school	8.51	6.34	
High school graduate/GED or equivalent	18.63	14.48	
College or above	72.87	79.18	
Marital status (%)			.7856
Married/living as married	69.95	71.09	
Single/divorced/widowed/never married	30.05	28.91	
Drinking status (%)			.0023
No	26.89	15.17	
Yes	73.11	84.83	
Hypertension (%)			.1411
No	76.96	82.4	
Yes	23.04	17.6	
Diabetes (%)			.391
No	93.95	95.7	
Yes	6.05	4.3	
Smoking status (%)			.4959
No	59.38	62.65	
Now	13.14	9.74	
Former	27.48	27.61	
Work activity (%)			.143
Vigorous	12.92	16.79	
Moderate	32.54	24.92	
Other	54.54	58.29	
Recreational activities (%)			.0592
Vigorous	33.85	41.18	
Moderate	32.17	34.46	
Other	33.98	24.36	
Energy (kcal)	14.34 ± 16.24	37.42 ± 49.83	<.0001
Total sugar (g)	1.05 ± 0.43	4.59 ± 5.20	.0075
Dietary fiber (g)	1.63 ± 4.37	4.92 ± 5.73	.0032
Fasting glucose (mg/dL)	92.17 ± 19.89	91.41 ± 21.78	.6982
2-h glucose (mg/dL)	114.85 ± 45.28	103.45 ± 32.51	.0514
FVC (mL)	3877.51 ± 963.60	4231.61 ± 1024.23	.0001
FEV1 (mL)	3029.94 ± 804.38	3291.24 ± 821.67	.0005
FVC%	101.79 ± 14.11	102.30 ± 13.48	.6884
FEV1%	91.42 ± 20.18	95.89 ± 17.39	.0105
FEV1/FVC	0.91 ± 0.19	0.94 ± 0.15	.0192

FEV1 = forced expiratory volume of 1 second, FVC = forced vital capacity, PIR = poverty income ratio.

### 3.2. Multivariate regression analysis

Multivariate regression analysis showed that carbohydrates was positively associated with FVC, and FEV1 (*P* < .05). Carbohydrate was positively associated with FVC% (*P* < .05). Moreover, dietary fiber was positively associated with FVC% and FEV1% (*P* < .05). Conversely, blood glucose was negatively correlated with lung function parameters. In particular, the 2-hour glucose was negatively correlated with FVC, FEV1 (*P* < .05). No significant association was found between FEV1/FVC and carbohydrates, total sugar, dietary fiber, fasting glucose, and 2-hour glucose were no significant difference (Table 2).

**Table 2:** Results of the multivariate regression analysis.

Variable	Lung function	Non-adjusted		Adjust I		Adjust II	
		β	95% CI	β	95% CI	β	95% CI
Carbohydrate	FVC	28.51***	16.09 to 40.92	16.01***	8.76 to 23.26	16.41*	3.94 to 28.87
	FEV1	20.30***	10.12 to 30.48	11.23***	5.39 to 17.06	15.62**	5.21 to 26.02
	FVC%	0.16	-0.02 to 0.33	0.19*	0.02 to 0.35	0.30*	0.03 to 0.57
	FEV1%	0.29*	0.05 to 0.52	0.15	-0.06 to 0.35	0.27	-0.07 to 0.62
	FEV1/FVC	0.00	-0.00 to 0.01	-0.00	-0.00 to 0.00	-0.00	-0.00 to 0.00
Total sugar	FVC	48.84**	17.46 to 80.21	31.84**	11.97 to 51.71	17.52	-13.53 to 48.56
	FEV1	37.06**	10.49 to 63.64	25.96**	8.13 to 43.79	15.25	-14.16 to 44.66
	FVC%	0.47*	0.04 to 0.90	0.46*	0.06 to 0.85	0.28	-0.37 to 0.93
	FEV1%	0.58*	0.05 to 1.11	0.45	-0.07 to 0.96	0.21	-0.66 to 1.09
	FEV1/FVC	0.00	-0.00 to 0.01	-0.00	-0.00 to 0.00	0.00	-0.00 to 0.00
Dietary fiber	FVC	24.44	-10.34 to 59.23	30.00**	8.92 to 51.07	22.20	-18.65 to 63.05
	FEV1	14.05	-14.11 to 42.22	24.64**	7.97 to 41.30	30.22	-3.04 to 63.47
	FVC%	0.75***	0.38 to 1.13	0.54**	0.14 to 0.93	1.07**	0.30 to 1.84
	FEV1%	0.67**	0.19 to 1.14	0.57*	0.06 to 1.08	1.25*	0.27 to 2.24
	FEV1/FVC	-0.00	-0.00 to 0.00	0.00	-0.00 to 0.00	0.00	-0.00 to 0.00
Fasting glucose	FVC	-2.03	-6.48 to 2.41	-3.62**	-6.30 to -0.94	-1.23	-5.24 to 2.78
	FEV1	-2.23	-5.86 to 1.39	-2.06	-4.24 to 0.12	-1.59	-4.98 to 1.79
	FVC%	-0.07*	-0.13 to -0.01	-0.12***	-0.17 to -0.06	-0.03	-0.12 to -0.06
	FEV1%	-0.14**	-0.22 to -0.06	-0.10**	-0.18 to -0.03	-0.02	-0.13 to 0.09
	FEV1/FVC	-0.00*	-0.00 to -0.00	-0.00	-0.00 to 0.00	0.00	-0.00 to 0.00
2-h glucose	FVC	-6.44***	-10.05 to -2.84	-3.40**	-5.57 to -1.23	-5.73**	-9.14 to -2.32
	FEV1	-5.14***	-7.96 to -2.32	-1.73*	-3.41 to -0.05	-3.27*	-6.13 to -0.41
	FVC%	-0.05*	-0.10 to -0.00	-0.07**	-0.11 to -0.02	-0.07	-0.15 to 0.01
	FEV1%	-0.06	-0.12 to 0.01	-0.05	-0.11 to 0.01	-0.05	-0.15 to 0.05
	FEV1/FVC	-0.00	-0.00 to 0.00	0.00	-0.00 to 0.00	0.00	-0.00 to 0.00

Non-adjusted: no adjustment covariates.

Adjust I: adjusted gender, age, race, education level, marital status, PIR.

Adjust II: adjusted gender, age, race, education level, marital status, PIR, body mass index, hypertension, diabetes, smoking status, drinking status, work activity, recreational activities, dietary energy.

FEV1 = forced expiratory volume of 1 s, FVC = forced vital capacity, PIR = poverty income ratio.

* *P* < .05.

** *P* < .01.

*** *P* < .001.

### 3.3. Spline smoothing and GAMM analysis

**Figure 1.**
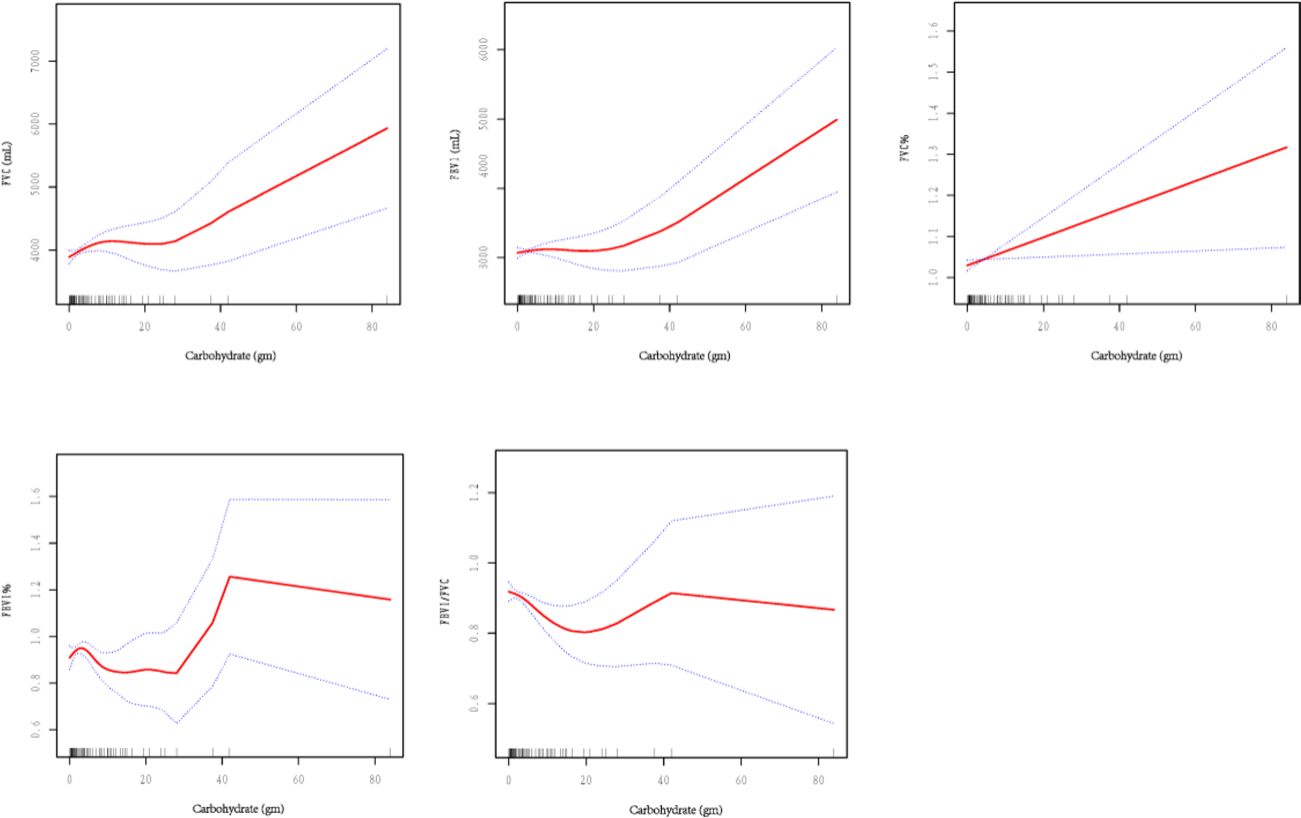
Relationship between carbohydrate intake and lung function.

Figure 1 illustrates the relationship between carbohydrates, total sugar, dietary fiber, fasting glucose, and 2-hour blood glucose level and lung function according to the smooth-curve fitting and GAMM. A linear relationship was noted between carbohydrates and FVC, FEV1, and FVC%. Conversely, a non-linear relationship was noted between carbohydrates and FEV1% and FEV1/FVC. A 1 g increase in the carbohydrate intake resulted in an FVC increase of 16.41 mL (95% CI = 3.94–28.87, *P* < .05), an FEV1 increase of 15.62 mL (95% CI = 5.21–26.02, *P* < .05), and an FVC% increase of 0.30% (95% CI = 0..03–0.57%, *P* < .05). A non-linear relationship with no inflection point was noted between carbohydrate intake and FEV1%, FEV/FVC. Conversely, an inflection point of 13.4 g was noted between the carbohydrate intake and FEV1% and FEV1/FVC. Below the inflection point, the carbohydrate intake had no significant correlation with FEV1% and a significantly negative correlation with FEV1/FVC. On the other hand, the carbohydrate intake above the inflection point was significantly positively correlated with FEV1%, but not with FEV1/FVC (Fig. 1).

**Figure 2.**
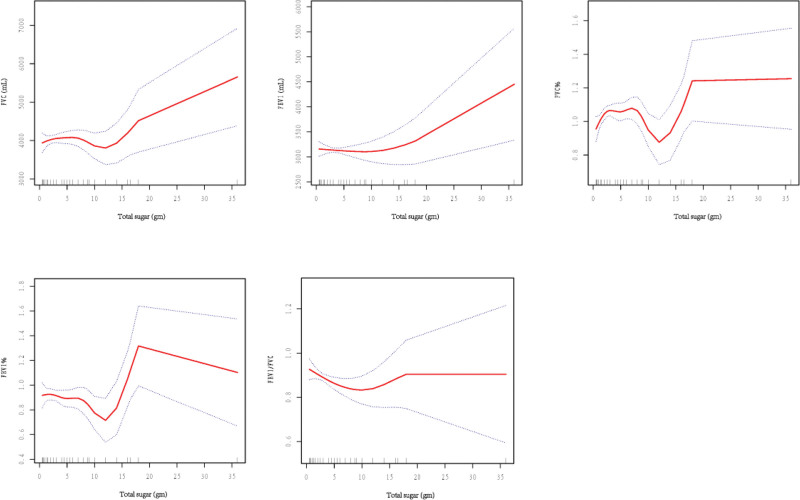
Relationship between total sugar intake and lung function.

A non-linear relationship was noted between total sugar intake and FVC, FEV1, FVC%, FEV1%, and FEV1/FVC (Fig. 2). The total sugar intake was more than 10 g, and the total sugar intake was positively correlated with FVC and FEV1. For every 1 g increase in total sugar intake, FVC increased by 46 mL (95% CI: 0.8438–91.7864, *P* < .05) and FEV1 increased by 57 mL (95% CI: 15.7896–100.1425, *P* < .05). An increase in the total sugar intake by 1 mg resulted in an FVC increase of 27.72 mL (95% CI: 5.75–49.69, *P* = .01), and an FEV1 increase of 26.69 mL (95% CI: 6.57–48.82, *P* = .01). Conversely, a negative relationship between total sugar and FEV1/FVC was noted below the inflection point of 7 g (β: -0.0135, 95% CI: -0.0240–0.0030, *P* < .05).

**Figure 3.**
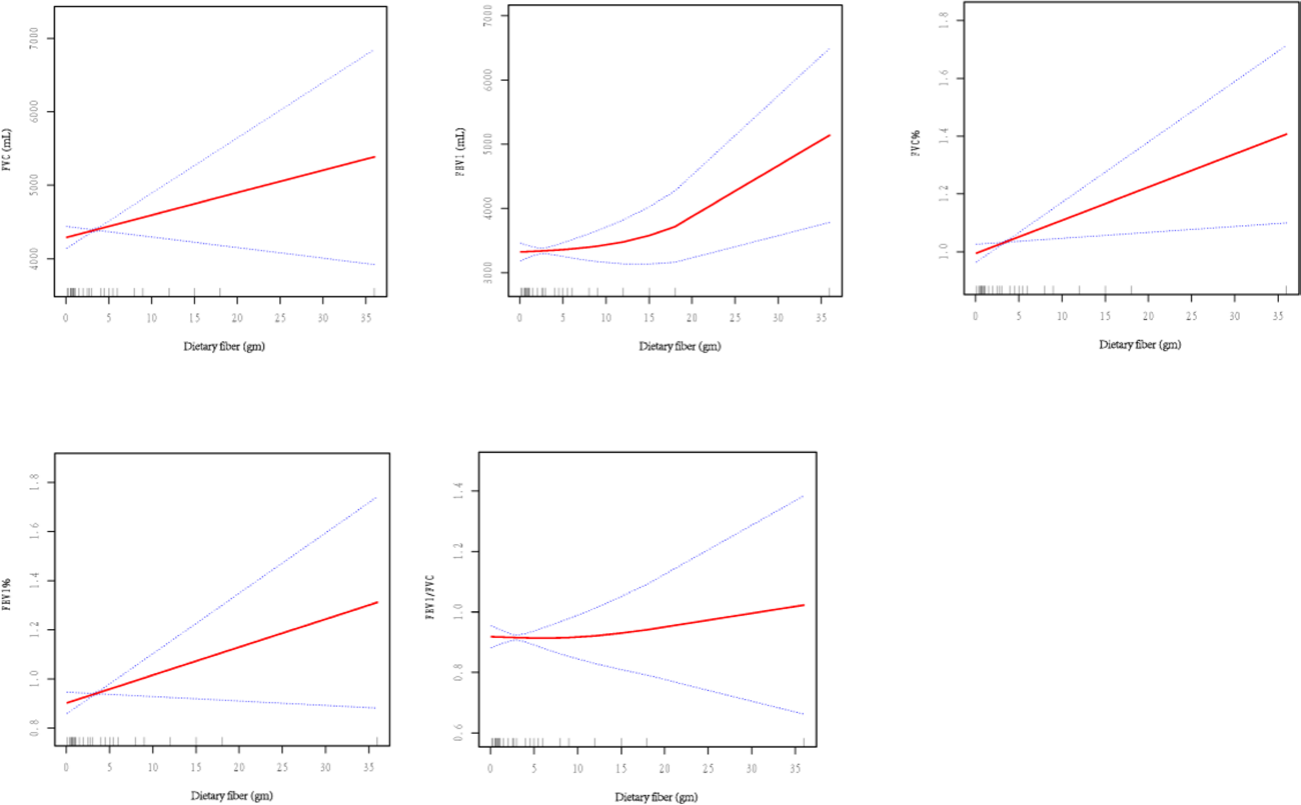
Relationship between dietary fiber intake and lung function.

A linear relationship was noted between dietary fiber and FVC%, FEV1% (Fig. 3). A 1 mg increase in the dietary fiber intake increased the FVC% by 1.07% (95% CI: 0.30–1.84%, *P* < .05) and FEV1% by 1.25% (95% CI: 0.27–2.24%, *P* < .05). The relationship between the dietary fiber intake and FVC and FEV1/FVC was non-linear with no significant inflection point. When dietary fiber intake was >9 g, there was a significant positive correlation between dietary fiber intake and FEV1 (β: 58.6286, 95% CI: 12.3576–104.8995, *P* < .05).

**Figure 4.**
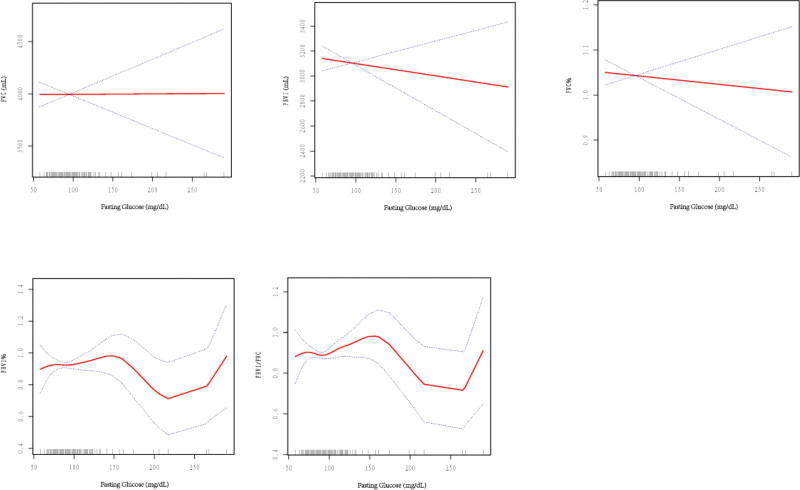
Relationship between fasting glucose and lung function.

A non-linear relationship was noted between fasting glucose and FVC, FEV1, FVC%, FEV1%, and FEV1/FVC. No obvious inflection point was observed for FVC, FEV1, FVC%, FEV1%, and FEV1/FVC (Fig. 4).

**Figure 5.**
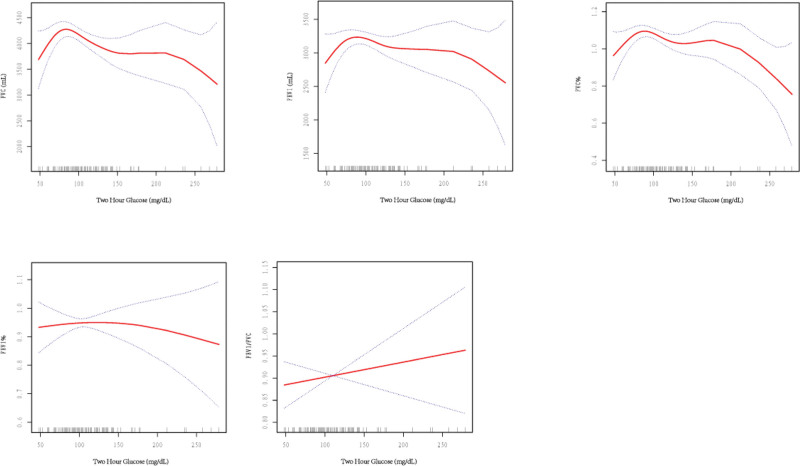
Relationship between 2-h glucose and lung function.

A linear negative correlation was observed between the 2-hour glucose and FVC, FEV1. An increase of 1 mg/dL in the 2-hour glucose resulted in a decrease in the FVC by 5.73 mL (95% CI: −9.14 to −2.32, *P* < .05) and the FEV1 by 3.27 mL (95%CI: −6.13 to −0.41, *P* < .05). The relationship between the 2-hour glucose and FEV1/FVC was non-linear with no significant inflection point (Fig. 5). Furthermore, relationship between 2-hour glucose and FVC% was noted inverted U shape, the inflection point was 67 mL (Fig. 5).

### 3.4. Subgroup analysis

In the male population, the intake of carbohydrates was significantly positively correlated with FEV1, and dietary fiber was also significantly positively correlated with FVC% and FEV1% (*P* < .05). However, a significant negative correlation was observed between the 2-hour glucose and FVC, FEV1, and FVC% (*P* < .05). In the female population, the carbohydrate intake was significantly positively correlated with FVC (*P* < .05). Conversely, a significant negative correlation was noted between the 2-hour glucose and FVC, FEV1, FVC% (*P* < .05, Table 3).

**Table 3:** Subgroup analysis according to gender.

Variable	Lung function	Male		Female	
		β	95% CI	β	95% CI
Carbohydrate	FVC	16.95	-3.57 to 37.48	20.35*	3.50 to 37.19
	FEV1	20.37*	2.67 to 38.06	10.67	-3.70 to 25.04
	FVC%	0.33	-0.07 to 0.72	0.27	-0.18 to 0.72
	FEV1%	0.46	-0.02 to 0.94	-0.01	-0.54 to 0.51
	FEV1/FVC	0.00	-0.00 to 0.00	-0.00	-0.00 to 0.00
Total sugar	FVC	36.56	-14.48 to 87.59	16.73	-38.69 to 72.14
	FEV1	23.75	-33.17 to 80.66	11.92	-34.30 to 58.14
	FVC%	0.40	-0.04 to 1.19	0.37	-1.03 to 1.76
	FEV1%	0.24	-1.08 to 1.57	0.08	-1.46 to 1.61
	FEV1/FVC	-0.00	-0.01 to 0.01	-0.00	-0.01 to 0.01
Dietary fiber	FVC	20.02	-40.80 to 80.84	31.12	-9.72 to 71.96
	FEV1	47.30	-4.03 to 98.62	13.69	-17.95 to 45.32
	FVC%	1.34**	0.40 to 2.28	0.71	-0.41 to 1.83
	FEV1%	1.92**	0.79 to 3.05	0.10	-1.09 to 1.29
	FEV1/FVC	0.01	-0.00 to 0.01	-0.00	-0.01 to 0.00
Fasting glucose	FVC	-1.10	-6.76 to 4.55	-1.24	-7.53 to 5.04
	FEV1	-1.41	-6.33 to 3.52	0.35	-5.04 to 5.74
	FVC%	-0.00	-0.11 to 0.11	-0.05	-0.22 to 0.12
	FEV1%	-0.03	-0.16 to 0.10	-0.02	-0.18 to 0.22
	FEV1/FVC	-0.00	-0.00 to 0.00	0.00	-0.00 to 0.00
2-h glucose	FVC	-18.65***	-27.75 to -9.56	-3.70**	-6.11 to -1.28
	FEV1	-10.12*	-18.65 to -1.59	-2.52*	-4.52 to -0.52
	FVC%	-0.21*	-0.38 to -0.03	-0.08*	-0.14 to -0.01
	FEV1%	-0.01	-0.37 to 0.18	-0.06	-0.13 to 0.00
	FEV1/FVC	0.00	-0.00 to 0.00	-0.00	-0.00 to 0.00

FEV1 = forced expiratory volume of 1 second, FVC = forced vital capacity, PIR = poverty income ratio. *P < .05. **P < .01. ***P < .001.

In participants aged between 20 and 40 years, a significant negative correlation was observed between the 2-hour glucose and FVC (*P* < .05). In participants aged above 40 years, carbohydrate intake was significantly positively correlated with FVC and FEV1 (*P* < .05). In addition, a significant negative correlation was noted between the 2-hour glucose and FVC, FEV1. Dietary fiber was significantly positively correlated with FVC, FEV1, and FEV1% (*P* < .05) while fasting blood glucose was significantly negatively correlated with FEV1 (*P* < .05, Table 4).

**Table 4:** Subgroup analysis according to gender.

Variable	Lung function	20–40 yr		>40 yr	
		β	95% CI	β	95% CI
Carbohydrate	FVC	9.65	-28.46 to 47.76	27.93**	11.41 to 44.44
	FEV1	15.06	-18.95 to 49.07	27.55***	13.70 to 41.40
	FVC%	-0.12	-0.88 to 0.63	0.31	-0.02 to 0.65
	FEV1%	-0.10	-0.98 to 0.77	0.33	-0.08 to 0.75
	FEV1/FVC	0.00	-0.00 to 0.00	0.00	-0.00 to 0.00
Total sugar	FVC	48.08	-28.98 to 125.15	13.37	-41.63 to 68.38
	FEV1	55.08	-10.03 to 120.19	8.80	-47.42 to 65.03
	FVC%	1.16	-0.50 to 2.81	-0.50	-1.44 to 0.45
	FEV1%	1.11	-0.61 to 2.82	-0.34	-1.73 to 1.05
	FEV1/FVC	0.00	-0.01 to 0.01	-0.00	-0.01 to 0.01
Dietary fiber	FVC	-5.26	-88.28 to 77.76	85.38**	29.04 to 141.72
	FEV1	31.44	-45.35 to 108.23	87.56***	44.70 to 130.41
	FVC%	0.15	-1.34 to 1.64	1.02	-0.04 to 2.08
	FEV1%	0.91	-1.27 to 3.09	1.26*	0.03 to 2.50
	FEV1/FVC	0.01	-0.00 to 0.02	0.00	-0.01 to 0.01
Fasting glucose	FVC	2.66	-6.92 to 12.25	-3.85	-9.32 to 1.62
	FEV1	3.86	-4.74 to 12.46	-5.58*	-10.23 to -0.92
	FVC%	0.07	-0.12 to 0.26	-0.01	-0.12 to 0.10
	FEV1%	0.12	-0.10 to 0.34	-0.05	-0.19 to 0.09
	FEV1/FVC	0.00	-0.00 to 0.00	-0.00	-0.00 to 0.00
2-h glucose	FVC	-4.88*	-8.92 to -0.84	-8.65***	-13.21 to -4.09
	FEV1	-1.75	-5.41 to 1.92	-6.86***	-10.24 to -3.48
	FVC%	-0.03	-0.12 to 0.06	-0.08	-0.18 to 0.02
	FEV1%	-0.03	-0.14 to 0.09	-0.05	-0.16 to 0.06
	FEV1/FVC	0.00	-0.00 to 0.00	0.00	-0.00 to 0.00

FEV1 = forced expiratory volume of 1 s, FVC = forced vital capacity, PIR = poverty income ratio. *P < .05. **P < .01. ***P < .001.

## 4. Discussion

In recent decades, exercise and diet have been recognized as major factors affecting health.^[21–23]^ Carbohydrates have an important role in disease development and intervention.^[24]^ Dietary fiber can affect the composition and abundance of microbes in the body, especially in the gut,^[25]^ and high fiber intake may have a range of health benefits for the host by affecting the gut microbiome.^[26]^ Carbohydrates consumed in moderation can reduce fatigue and improve well-being,^[27]^ mood, mental health, and cognition.^[28,29]^ However, some studies have shown that a diet high in sugar consumption can have adverse effects on mental health.^[30,31]^ Increased intake of refined grains was linked with an increased risk of metabolic disease,^[32]^ while an increased intake of whole grains was linked with reduced risk.^[33]^ A diet high in fiber was also found to have a beneficial effect on chronic kidney disease.^[34]^ However, there are few studies on the effects of carbohydrates on respiratory health, and the effects of carbohydrates on lung function are not clear.

In our study, we evaluated the impact of the type and amount of carbohydrate intake on lung function. The increased intake of carbohydrates and dietary fiber increased the FVC and FEV1. Consistent with the finding by Corrine Hanson et al, we also found that the intake of dietary fiber increased lung function.^[35]^ In contrast, 2-hour glucose was negatively associated with lung function. The 2-hour glucose test provides valuable information on glucose tolerance and pancreatic islet function. Deficiencies in pancreatic function may lead to malabsorption and nutritional deficiencies, thickened mucus secretions, increased susceptibility to infections, and systemic inflammation. All these factors may influence lung function and increase the risk of developing lung disease.

We also noted a stronger association between carbohydrate intake and lung function in men and individuals over 40 years old. Sex hormones can influence glucose tolerance and pancreatic function. Moreover, age-related changes within the pancreas can contribute to the development of insulin resistance, altered glucose metabolism, and ultimately lung function.

Low carbohydrate diets have long been thought to be beneficial in the treatment of various diseases including epilepsy,^[36]^ diabetes, cancer, and gastrointestinal, pulmonary, and cardiovascular diseases.^[37–40]^ A high carbohydrate diet can strain the pancreas by increasing insulin production, potentially leading to insulin resistance and inflammation, which may negatively impact lung function.^[41]^ However, a diet low in carbohydrate intake often results in an increased intake of fat and/or protein, which increases the risk of hyperlipidemia and hypercholesterolemia.^[42]^ In this study, we identify the optimal carbohydrate intake that had the least impact on lung function.

This study has some limitations that have to be acknowledged. The cross-sectional design of our study only allowed us to collect information on carbohydrate intake at a single point in time. However, prolonged exposure to carbohydrates is necessary to produce long-lasting effects on human health. Therefore, longitudinal studies are necessary to confirm our findings. Moreover, we did not elucidate the pathological mechanism that leads to poor lung function in patients with excessive carbohydrate intake. Finally, it is also important to note that several other factors such as genetics, other dietary factors, and exercise can also influence carbohydrate metabolism and lung function. These factors were not taken into account in this study.

## 5. Conclusion

Carbohydrates and dietary fiber intake were positively associated with FVC and FEV1. Conversely, the 2-hour glucose was negatively associated with lung function. The association between carbohydrates and lung function was more significant in men and individuals above the age of 40 years. The findings of this study could be used to develop dietary interventions that can enhance lung function and reduce the risk of developing lung disease.

## Acknowledgments

We would like to thank TopEdit (www.topeditsci.com) for the English language editing of this manuscript.

## Author contributions

**Conceptualization:** Yin Xu, Wei Wei, Ning Li.

**Data curation:** Yin Xu, Xinmei Wang, Guofeng Wang, Wei Wei.

**Formal analysis:** Yin Xu, Xinmei Wang, Guofeng Wang, Wei Wei.

**Investigation:** Guofeng Wang.

**Methodology:** Yin Xu, Wei Wei, Ning Li.

**Resources:** Guofeng Wang.

**Software:** Yin Xu, Xinmei Wang.

**Supervision:** Yin Xu, Xinmei Wang, Guofeng Wang, Wei Wei, Ning Li.

**Validation:** Yin Xu, Xinmei Wang, Guofeng Wang, Wei Wei, Ning Li.

**Visualization:** Yin Xu, Xinmei Wang, Wei Wei.

**Writing – original draft:** Yin Xu, Xinmei Wang, Guofeng Wang, Wei Wei, Ning Li.

**Writing – review & editing:** Yin Xu, Guofeng Wang, Wei Wei, Ning Li.
